# Novel synthetic RORγ agonist compounds as a potential anti-tumor therapeutic approach

**DOI:** 10.1186/2051-1426-2-S3-P194

**Published:** 2014-11-06

**Authors:** Xiao Hu, Xikui Liu, Rodney Morgan, Jacques Moisan, Ling-Yang Hao, Yahong Wang, Brian Sanchez, Charles Lesch, Dick Bousley, Clark Taylor, Thomas Aicher, Peter Toogood, Weiping Zou, Gary Glick, Laura Carter

**Affiliations:** 1 Lycera Corp., Ann Arbor, MI, USA; 2 Dept. of Surgery, Immunology, Biology, University of Michigan, Ann Arbor, MI, USA

## Introduction

Selective enhancement (or activation) of the immune system by novel small molecules may be a potential therapeutic approach for the treatment of cancer. RORγt (**R**etinoic Acid Receptor-related **o**rphan **r**eceptor) is the key transcription factor for the development of CD4^+ ^Th17 cells, CD8^+ ^Tc17 cells and IL-17^+ ^innate immune cells including γδ T cells. A member of the nuclear receptor superfamily, RORγ modulates the expression of cytokines, chemokines and their receptors to induce a pro-inflammatory environment. RORγ can interact with other lineage-associated transcription factors resulting in developmental plasticity which reinforces immunity and limits immunosuppressive mechanisms. These activities suggest that the activation of RORγ may enhance anti-tumor immune responses and Th17 and Tc17 cells have been reported to have potent anti-tumor effects in vivo.

## Results

We have discovered a series of synthetic RORγ agonist compounds that enhance the activity of an RORγ-dependent reporter and increase IL-17A, IL-17F, IL-22 and GM-CSF production by murine and human T cells. CD4^+ ^T cell stimulation with cytokine cocktails including TGFβ in vitro can generate Th17 and Treg cells; the addition of RORγ agonists decreases the expression FOXP3 while concomitantly increasing IL-17 and GM-CSF mRNA expression. In addition, stimulation of T cells in the presence of RORγ agonist compounds generates effector T cells that resist PD-1/PD-L1-mediated inhibition of proliferation and cytokine production. Taken together, the enhanced production of cytokines, decreased generation of Treg cells and resistance to PD-1 checkpoint inhibition supports RORγ agonists having potential anti-tumor activities. Indeed, OVA-specific CD8^+ ^T cells activated in vitro with RORγ agonist compounds reduced growth of OVA-expressing EG7 and B16F10 tumors more effectively than cells stimulated without RORγ agonist. Flow cytometric analysis of tumors over time showed increased numbers of agonist treated cells present as tumor infiltrating lymphocyte (TILs). A higher percentage of these TILs were IL-17^+ ^with increased mean fluorescent intensity for IL-17. Agonist treated TILs had increased expression of CD62L and decreased expression of PD-1 suggesting that agonist treatment preserves a less differentiated, more central memory phenotype that may be less susceptible to checkpoint inhibition and have increased survival capacity.

## Conclusions

RORγ agonist compounds enhance immune-associated anti-tumor pathways, decrease immunosuppressive mechanisms and induce effector T cells which decrease tumor growth. Immune enhancement by RORγ agonists may therefore represent a unique anti-tumor approach which could also complement other immunotherapy approaches.

